# Rapid delineation of *Acinetobacter baumannii* dose responses to zosurabalpin based on machine learning-assisted interpretation of bacterial nanomotions

**DOI:** 10.1128/spectrum.04064-25

**Published:** 2026-06-30

**Authors:** Marta Pla Verge, Jan Winnicki, Amanda Luraschi-Eggemann, Grzegorz Jozwiak, Katja Fromm, Laura Munch, Gino Cathomen, Caspar Vogel, Florian Schwanke, Danuta Cichocka, Alexander Sturm

**Affiliations:** 1 Resistell AG 727115 , Muttenz, Switzerland; Boston Medical Center, Boston, Massachusetts, USA

**Keywords:** nanomotions, zosurabalpin, antibiotic susceptibility test, machine learning, AMR, AST, AI, diagnostics, blood culture, antibiotic

## Abstract

Carbapenem-resistant *Acinetobacter baumannii* (CRAB) represents a major clinical threat, underscoring the urgent need for both new antibiotics and rapid antimicrobial susceptibility testing (AST) methods that can inform treatment. Here, we describe a susceptibility assessment that combines nanomotion technology with machine learning (ML) to rapidly classify responses to zosurabalpin (ZAB), the first-in-class antibiotic targeting the LptB₂FGC complex of the lipopolysaccharide transport pathway in *A. baumannii*. Nanomotion recordings from five *A. baumannii* strains spanning a wide range of minimum inhibitory concentrations (MICs) were measured at multiple ZAB concentrations. A logistic regression ML model was trained using a labeling strategy that categorized recordings as susceptible or non-susceptible based on strain MICs obtained by reference methods and antibiotic concentrations measured. This model, built on four quantile features, achieved >90% accuracy compared with susceptibility responses. These results highlight four key advances: (i) nanomotion-based AST can deliver reliable phenotypic classification for new antibiotics before clinical breakpoints are available, (ii) accurate results can be obtained within only 2 h, (iii) the method extends nanomotion’s applicability to high-priority pathogens, such as CRAB, and (iv) the nanomotion response can reflect early cellular changes that precede a measurable decline in viability, suggesting that susceptibility may be detected before killing becomes apparent by conventional methods. Together, this work establishes nanomotion-ML AST as a promising diagnostic platform to support the evaluation and future integration of novel drugs into clinical practice, advancing next-generation rapid AST workflows against critical multidrug-resistant pathogens.

**IMPORTANCE:**

This study demonstrates a rapid, nanomotion-based approach with potential to inform antibiotic susceptibility assessment for *Acinetobacter baumannii* infections. By combining nanomotion technology with quantitative machine-learning-assisted signal analysis, susceptibility to the novel antibiotic zosurabalpin, which is currently in clinical development, could be determined within 2 h. The assay reliably distinguished susceptible from non-susceptible phenotypes across strains with different MICs and in both standard and horse serum-supplemented media. Notably, the nanomotion signature reflected early cellular responses that preceded a measurable decline in viability by conventional methods. Together, these features provide rapid phenotypic insights that may support future development of timely, targeted therapy decisions. The findings highlight nanomotion-based antimicrobial susceptibility testing as a promising tool to accelerate diagnostic readiness for new antibiotics and improve treatment management in infections caused by multidrug-resistant *A. baumannii*.

## INTRODUCTION

The continuous rise of antimicrobial resistance (AMR) is one of the greatest threats to global health, representing a leading cause of death worldwide and steadily reducing the number of effective antibiotics available to treat bacterial infections, even for newly approved therapeutics, for which resistance is reported within 1 year of market approval (1–3).

*Acinetobacter baumannii* is a particularly problematic Gram-negative pathogen within the ESKAPE group due to its remarkable ability to acquire resistance through antibiotic target mutations, horizontal gene transfer of antibiotic-hydrolyzing enzymes, efflux pump changes or altered cellular membrane permeability, and disseminate in patient settings, causing severe hospital-associated infections such as pneumonia and bloodstream infections (4–9). The global spread of multidrug-resistant clones, including carbapenem-resistant *A. baumannii* (CRAB) (10, 11), has led the World Health Organization to rank this species in the critical priority group, underlining the urgent need for novel treatments and rapid phenotypic diagnostic tools (12). Administration of reserve antibiotics can only happen after susceptibility to standard treatments has been ruled out, and efficacy has been demonstrated through *in vitro* testing (13, 14). This two-tiered verification process is designed to prevent overuse and preserve the clinical utility of novel compounds. However, workflows based on conventional growth-dependent antibiotic susceptibility testing (AST) methods, such as disk diffusion or broth microdilution, are inherently slow and often incompatible with the urgency of treating critically ill patients.

Current international standard methods for AST, such as broth microdilution or disk diffusion assays, typically require 18–24 h to deliver results. Automated AST platforms provided by diagnostic companies often rely on predefined antibiotic panels in cartridge systems (15). Due to clinical development timelines and regulatory constraints, novel antibiotics are usually integrated into such systems only years after their initial approval. For instance, the last-resort drug combination ceftazidime–avibactam, approved by the FDA in 2015, was integrated into the widely used automated AST platform Vitek2 after 2 years (16, 17), while cefiderocol, approved in 2019, has not yet been introduced. The delay in introducing novel drugs into fast, automated AST platforms may lead to inadequate therapies and is considered one of the main reasons for rising rates of resistance to novel antibiotics (2, 18). It frequently forces clinicians to administer broad-spectrum antibiotics empirically, delaying targeted therapy and risking mismatches with the actual resistance profile of the infecting pathogen. Such delays contribute to poor patient outcomes. In *A. baumannii*, inappropriate or delayed treatment has been especially detrimental, accelerating the rise of resistant clones and fueling increasing AMR rates in recent years (19, 20). For countermeasures, it is important to obtain rapid, reliable susceptibility information from an AST (21).

**Fig 1:**
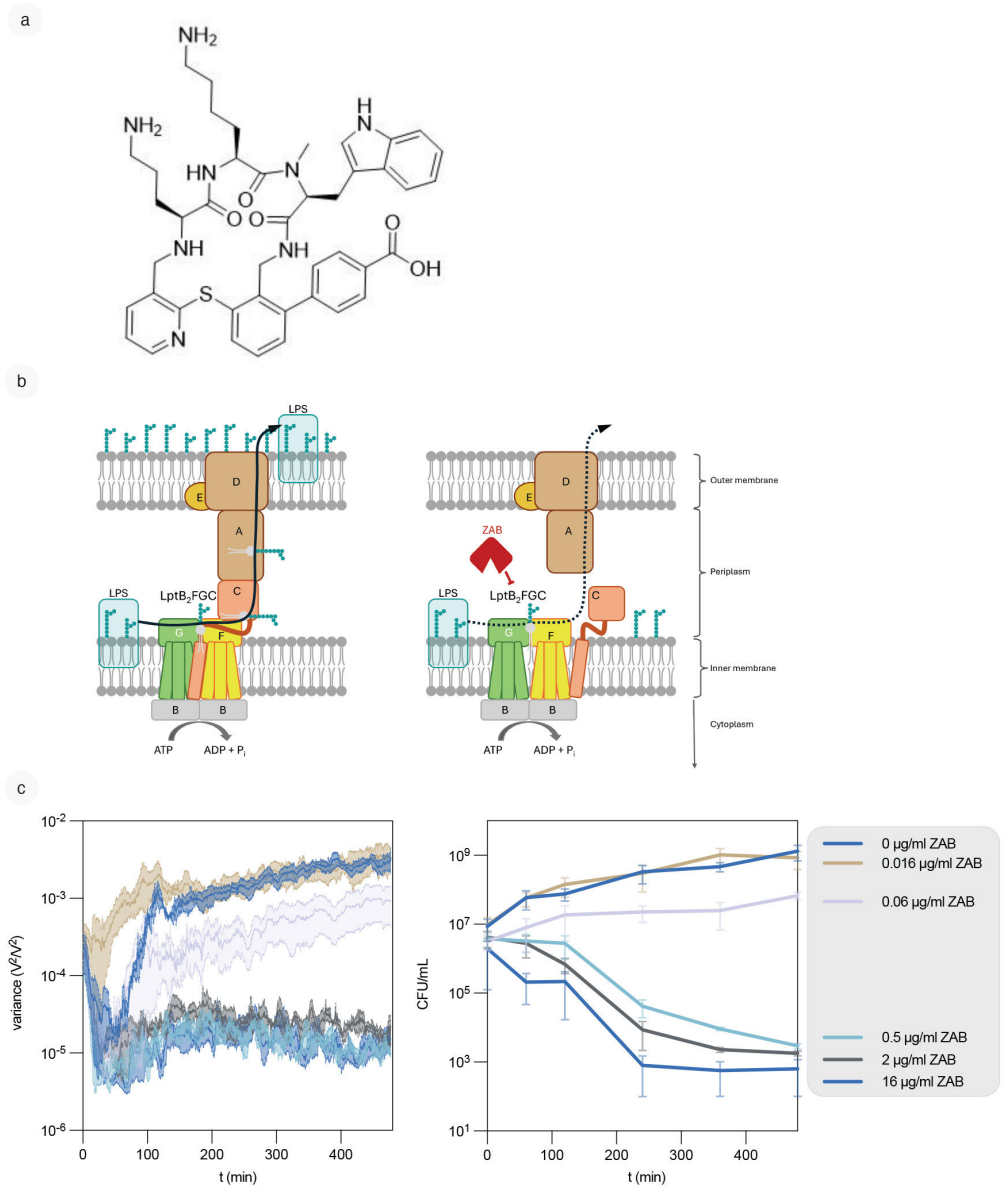
Mechanism of ZAB action and nanomotion response of *A. baumannii*. (**a**) Chemical structure of ZAB. (**b**) Mechanism of LPS transport and its inhibition by ZAB. Under normal conditions (left), the LptB₂FGC complex in the inner membrane transports LPS to the outer membrane, maintaining membrane integrity. When ZAB binds to LptB₂FGC (right), transport is blocked, disrupting outer membrane assembly and bacterial viability. (**c**) Left panel: 8 h nanomotion recordings of *A. baumannii* ATCC 17,978 (MIC = 0.06 µg/mL) in HoS-CAMHB exposed to increasing concentrations of ZAB (0, 0.016, 0.06, 0.5, 2, and 16 µg/mL). Data represents triplicate measurements (mean ± SEM). Right panel: Triplicate time-kill assays performed in parallel to nanomotion recordings in HoS-CAMHB over 8 h, with CFU quantification performed at 0, 1, 2, 4, 6, and 8 h (mean ± SEM). Concentrations ≥ 4 × MIC produced bactericidal activity, consistent with previous observations (23).

The macrocyclic peptide zosurabalpin (ZAB) (Fig. 1a) is a recently developed clinical-stage bactericidal antibiotic that targets the lipopolysaccharide (LPS) transport pathway in *A. baumannii* by inhibiting the LptB_2_FGC complex (Fig. 1b) and disrupting its transfer from the inner to the outer membrane (22–24). The new mode of action (MoA) of ZAB is particularly noteworthy, as it reduces the degree of cross-resistance to clinically used antibiotic classes (25). By specifically targeting *A. baumannii* and not LptB_2_FGC complexes of other Gram-negative species, ZAB provides advantages over broad-spectrum agents. Its pathogen-specific activity minimizes disruption of the host microbiome, reducing the risk of secondary infections and dysbiosis, and may slow the emergence and spread of antimicrobial resistance (26).

Despite previous *in vitro* potency analyses, there is yet a lack of clinical breakpoints, antibiotic concentrations determined by the European Committee on Antimicrobial Susceptibility Testing (EUCAST), or the Clinical and Laboratory Standards Institute (CLSI) (23). Clinical breakpoints are determined based on epidemiological cut-off values, minimum inhibitory concentration (MIC) distributions, and clinical outcome data. They serve as thresholds to classify isolates as susceptible or resistant, thereby guiding whether a given antibiotic should be used or avoided. For ZAB, clinical breakpoints will be established only after the completion of phase III clinical trials and their incorporation into official guidelines. Until then, we avoid the terminology of “resistant” as it is not yet defined what MIC refers to clinical resistance. Instead, we use “non-susceptible”—phenotype or “non-susceptible”—response when nanomotions were measured above the strain-specific MIC.

Nanomotion technology has been presented as a rapid, phenotypic, and growth-independent alternative method for susceptibility testing (27–30). The measurement of nanoscale vibrations of living cells offers a shorter time to results than traditional methods (15) in fast-growing bacteria such as the Enterobacterales group (27, 31) and slow-growing bacteria like *Mycobacterium tuberculosis* (32–34) and has been validated in clinical settings (27, 35, 36). The signal variance during drug exposure can be considered an indicator of bacterial drug susceptibility. A clinically applicable nanomotion AST covering the heterogeneous nanomotion responses of clinical isolates demands implementation of machine learning (ML)-trained classification models (27, 30, 33, 37). Recently, a study explored the nanomotion of *A. baumannii,* showing a correlation between variance amplitude and metabolic activity by comparing nutrient-rich with nutrient-limited media. Antibiotic susceptibility correlated with smaller oscillations (38), as initially reported for nanomotions and *Escherichia coli* (28, 39).

In this work, we develop the first growth-independent susceptibility assessment for ZAB and *A. baumannii,* using nanomotion technology and ML techniques to classify susceptibility phenotypes in 2 h. Five strains with a broad range of MICs were tested across multiple ZAB concentrations to capture susceptibility responses in relation to their MICs. For the training of the algorithm, we labeled each experiment as non-susceptible (NS) or susceptible (S) according to the measurement concentration in relation to the strain-specific MIC. In this regard, the algorithm did not rely on predefined breakpoints and was capable of creating a model that distinguishes S and NS responses depending on the MIC and measurement concentration. Our findings broaden the applications of high-resolution nanomotion as a phenotypic AST platform as part of the pipeline toward next-generation rapid AST methods.

## RESULTS

### Correlation between nanomotion dynamics and conventional time-kill kinetics for zosurabalpin

To investigate whether nanomotion responses reflected conventional bactericidal kinetics, *A. baumannii* ATCC 17978 reference strain (MIC = 0.06 µg/mL) was exposed to increasing concentrations of ZAB (0, 0.016, 0.06, 0.5, 2, and 16 µg/mL) and monitored in parallel with nanomotion recordings and standard time-kill assays for 8 h. Experimental conditions were designed based on the methodology described by Zampaloni and coworkers (23), who reported bactericidal activity of ZAB at concentrations ≥ 4× MIC in conventional killing assays.

Nanomotion recordings were performed in triplicate in 20% heat-inactivated horse-serum-supplemented cation-adjusted Mueller-Hinton broth (HoS-CAMHB) at 37°C (Fig. 1c). Similar to previously established protocols (27, 30), bacteria were recovered from spiked positive blood cultures (PBCs), immobilized on individual cantilevers (Fig. S1a), and the recordings consisted of a 30-min baseline measurement of nanomotion activity in HoS-CAMHB, followed by the exposure to different ZAB concentrations for 8 h.

Under near-identical conditions, standard time-kill experiments were conducted with colony forming unit (CFU) quantification at 0, 1, 2, 4, 6, and 8 h (Fig. 1c). They differed as bacteria were not attached to a cantilever surface, and the inoculum was ~10^6^ bacteria. At sub-MIC (0 and 0.016 µg/mL). Bacterial growth was observed in time-kill assays for which we in parallel also observed a progressive increase in nanomotion variance. At the reported MIC = 0.06 µg/mL (23), the increase of the variance was less pronounced; however, we also observed a slight increase in the CFU count over time indicating that under our conditions the growth is not entirely inhibited. Trailing was reported for *A. baumannii* strains before. Importantly, nanomotion increase or a high nanomotion signal in general cannot be equated to growth, as was shown in previous publications for non-growing antibiotic-tolerant *E. coli*, as well as *M. tuberculosis* in which bacteria were kept in non-growing conditions (28, 32, 33, 40). In contrast, concentrations ≥4× MIC (0.5, 2, and 16 µg/mL) produced an early reduction in the nanomotion signal, accompanied by a decrease in CFU counts, which was especially notable after 2 h of ZAB exposure. Based on these results, the variance of the nanomotion signal is mainly reflecting viability for ZAB-exposed *A. baumannii* and less growth inhibition reflected by MIC values. For the establishment of a competitive rapid AST, we decided to not exceed 90-min drug exposure corroborated by previous studies on Enterobacterales and *P. aeruginosa* (27, 30).

### Zosurabalpin nanomotion-based AST experimental design

**Fig 2:**
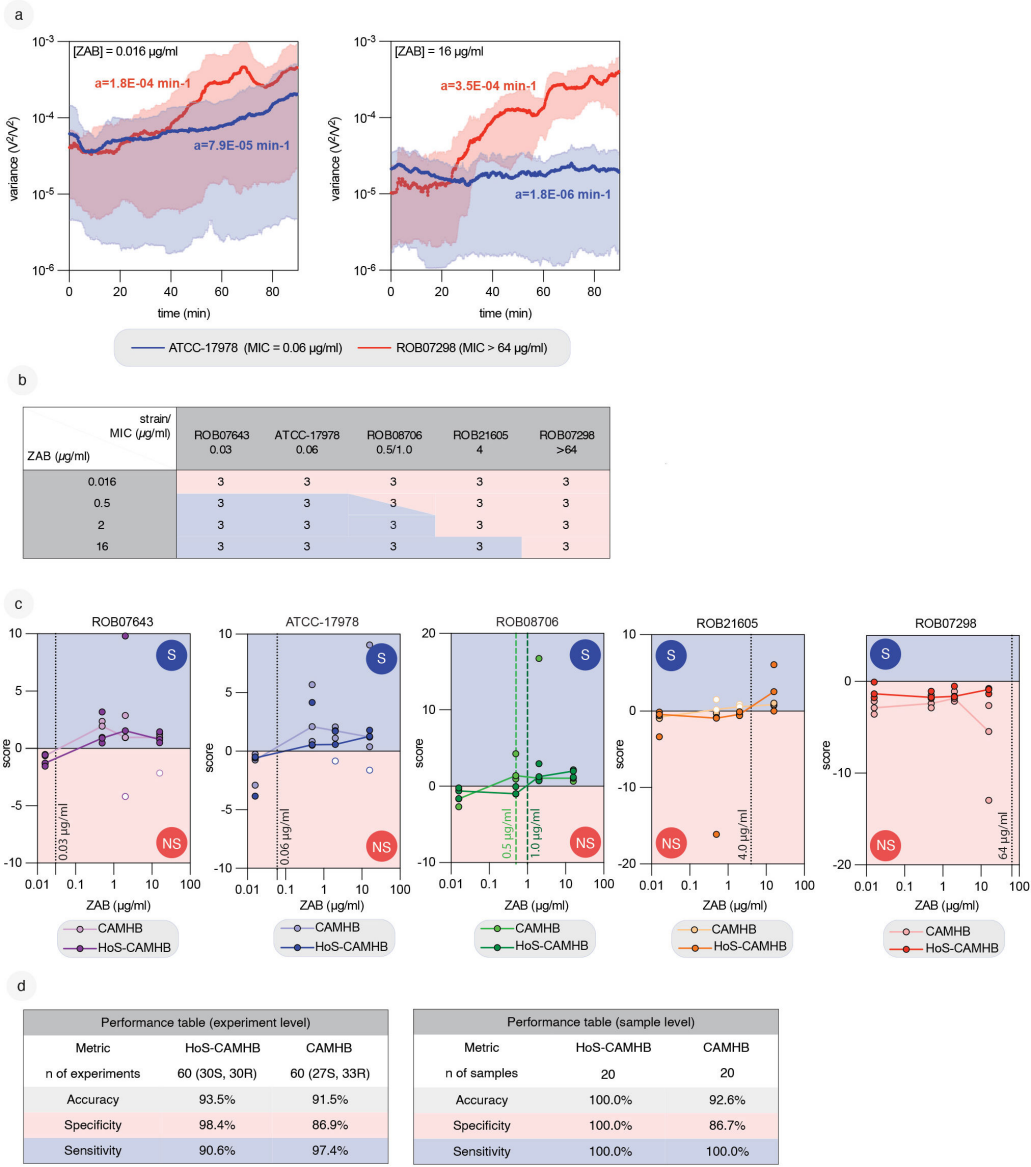
Performance of the nanomotion-based classification model across ZAB concentrations and media. (**a**) Running median of nanomotion signal variance after exposure to 0.016 (left) or 16 µg/mL (right) ZAB in *A. baumannii* ATCC 17978 (MIC = 0.06 µg/mL, blue) and ROB07298 (MIC > 64 µg/mL, red) in CAMHB. Data represent the mean ± range of three biological replicates after isolation from a spiked positive blood culture. (**b**) Experimental setup showing *A. baumannii* strains with respective MICs and ZAB measurement concentrations. Numbers indicate replicates per condition (60 experiments in CAMHB and 60 in HoS-CAMHB). Experiments were labeled non-susceptible (red) when [ZAB] < MIC and susceptible (blue) when [ZAB] ≥ MIC. Strain ROB08706 had MICs of 0.5 µg/mL in CAMHB and 1.0 µg/mL in HoS-CAMHB. (**c**) Mean scores from 500 repeated cross-validations of the four-feature classification model are shown for five *A. baumannii* strains across ZAB concentrations. Vertical dotted lines indicate MICs. Lighter shades correspond to CAMHB and darker shades to HoS-CAMHB. Lines connect median scores across replicates, and empty circles mark misclassified experiments. (**d**) Model performance based on 500 repeated cross-validations at the experiment level (left) and sample level (median of triplicates, right) in both media, benchmarked against reference labels according to ZAB concentration and MIC. Accuracy = overall concordance; specificity = correct non-susceptible classifications ([ZAB] < MIC); sensitivity = correct susceptible classifications ([ZAB] ≥ MIC).

We evaluated the nanomotion response of five *A. baumannii* strains (four wild-type strains and one laboratory-generated resistant mutant) with varying MICs for ZAB. Here, however, samples were analyzed using the previously established 2-h measurement protocol (27), comprising a 30-min baseline recording in cation-adjusted Mueller-Hinton broth (CAMHB) or HoS-CAMHB, followed by 90 min of ZAB exposure rather than the 8-h recording used above. Consistent with previous observations in Enterobacterales exposed to antibiotics with different MoAs, sub-MIC conditions produced a progressive increase in variance over time (27, 28, 31). In contrast, exposure to supra-MIC concentrations typically resulted in a stagnant response rather than a sharp decrease in variance, suggesting that bacterial cells did not undergo rapid lysis, in contrast to previously observed nanomotion responses of *E. coli* to membrane-disrupting agents such as polymyxins (40). The reference strain *A. baumannii* ATCC 17978 (MIC = 0.06 µg/mL) showed an increase in variance during exposure to sub-MIC concentrations of 0.016 µg/mL ZAB, but a stable, flattened response at 16 µg/mL, well above the MIC (Fig. 2a, blue). By comparison, the laboratory-generated *lptF* mutant ROB07298 resistant to ZAB (MIC >64 µg/mL) exhibited increasing variance at both concentrations, with a more pronounced effect at the higher concentration tested, which presumably inflicts higher stress levels (Fig. 2a, red). To investigate whether the observed increase in variance was associated with bacterial replication or biomass accumulation on the cantilever, microscopy images of all five tested strains were acquired under both susceptible (S) and non-susceptible (NS) conditions at the time of bacterial attachment and at the end of nanomotion recording. Only minimal changes in biomass were observed throughout the experiment, including under NS conditions (Fig. S1a), suggesting that the increase in variance is more likely linked to bacterial physiological activity and stress responses rather than to cell replication or biomass accumulation on the cantilever. This is consistent with previous studies of non-growing *E. coli* showing high variance signals (40).

Subsequently, we measured all five *A. baumannii* strains at four ZAB concentrations (0.016, 0.5, 2, and 16 µg/mL) that lay within the MIC range of those strains. Experiments were performed in triplicates, each replicate consisting of a bacterial pellet obtained from a spiked PBC. Each pellet was measured in parallel (i) with and (ii) without 20% heat-inactivated horse serum supplemented CAMHB in the nanomotion measurement chamber. According to CLSI recommendations, serum addition improved MIC determination by minimizing trailing effects and skipped wells in broth microdilution assays (23, 41). These experiments also enabled evaluation of the serum’s influence on nanomotion platform performance due to the increased viscosity of the media surrounding the cantilever. The resulting data set comprised 60 experiments in CAMHB and 60 in HoS-CAMHB.

### Zosurabalpin specificity to *A. baumannii*

Since ZAB has been reported to display highly specific activity against *A. baumannii* (23, 26), we additionally evaluated the nanomotion response of *E. coli* ATCC 25922 upon exposure to three ZAB concentrations (Fig. S1b). In agreement with the reported narrow-spectrum profile of ZAB, no measurable nanomotion alterations were detected for *E. coli* under any tested concentrations. To further illustrate that nanomotion responses vary depending on the antibiotic and its concentration, *E. coli* ATCC 25922 was exposed to ceftriaxone (CRO, MIC_CRO_ = 0.047 µg/mL, Fig. S1c) and meropenem (MEM, MIC_MEM_ = 0.016 µg/mL, Fig. S1c), two β-lactam antibiotics belonging to different subclasses, targeting different PBPs and with distinct pharmacodynamic properties and killing kinetics. For experimental consistency, antibiotic concentrations were selected to match similar MIC-relative ratios used in this study (0.25×, 8×, and 267× MIC). Sub-MIC concentrations present higher positive variance slopes than 8× MIC concentrations for both antibiotics and negative slopes at 267× MIC. Distinct nanomotion patterns were observed following exposure to CRO and MEM, supporting the notion that the temporal nanomotion response is strongly influenced by the antibiotic mechanism of action and associated cellular effects.

### Classification model training using machine learning

To develop a rapid, high-performing growth-independent AST based on nanomotion patterns of *A. baumannii*, we implemented an ML-based classification model trained on features extracted from the nanomotion signal during 90 min of ZAB exposure. A labeling strategy was applied in which each experiment was categorized as “susceptible” or “non-susceptible” according to the reference MIC of the corresponding strain and the ZAB concentration used in the measurement. For each experiment, samples were measured in parallel at four concentrations. In the absence of published clinical breakpoints, measurements performed with ZAB concentrations below the reference MIC were labeled as non-susceptible, whereas those performed at or above the MIC were labeled as susceptible (Fig. 2b). This classification is based on the ratio between the measurement concentration and the strain-specific MIC and therefore represents a relative, internally consistent definition rather than a clinical categorization based on established breakpoints. Reference MICs were concordant between CAMHB and HoS-CAMHB for all strains except ROB08706, where the MIC increased marginally from 0.5 to 1 µg/mL in HoS-CAMHB. This shift resulted in the relabeling of three experiments from susceptible to non-susceptible (Fig. 2b).

For feature extraction, the nanomotion signal with a sampling frequency of 60 kHz was divided into time segments for which the power spectral density was calculated. The features were obtained from the quantile spectra, 10th to 90th percentiles, including derived features describing relationships between quantiles across frequencies and time intervals (27, 37), resulting in a large pool of features (*n* = 311,040). The forward feature selection process allowed the identification of the most relevant features for the discrimination between the susceptible and non-susceptible labeled groups for each condition (with or without horse serum). From the initial pool of features, we did not select more than four features for the final model to avoid overfitting. The features with the highest improvement in the accuracy were selected and combined with a logistic regression model, resulting in a numerical value defined as ‘score’ for each recording. Negative scores correspond to the non-susceptible classification and are supposed to correctly identify experiments performed with ZAB concentrations below the MIC of the tested strain; whereas positive scores correspond to susceptible classification presumed for experiments with ZAB concentrations at or above the MIC of the strain tested.

**Fig 3:**
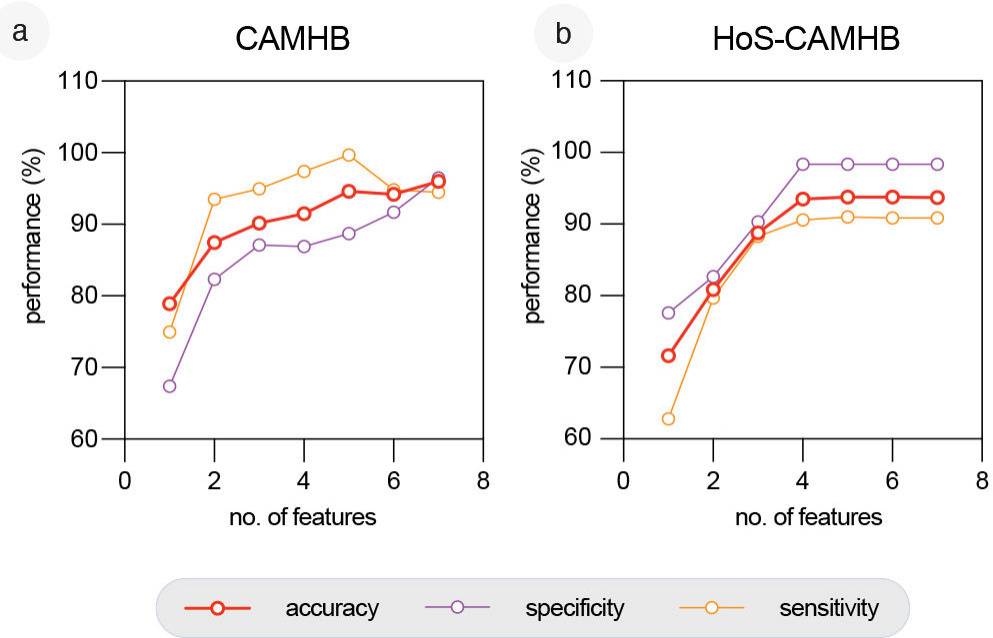
Performance of the classification model as a function of feature addition. Fluctuations in accuracy, sensitivity, and specificity are shown across iterations of feature inclusion, based on the cross-validation mean scores against the artificial labels at the experiment level (susceptible or non-susceptible phenotypic response) for CAMHB (**a**) and HoS-CAMHB (**b**).

Across all tested strains, nanomotion scores correlated with the ZAB measurement concentrations in accordance with the respective MICs. For example, *A. baumannii* ATCC 17,978 (MIC = 0.06 µg/mL) showed, as expected, scores <0 when [ZAB] < MIC, and scores >0 when [ZAB] > MIC, with the exception of two misclassified replicates at [ZAB] = 2.0 and 16 µg/mL in HoS-CAMHB (Fig. 2c, second plot). In this study, we used threefold cross-validation to evaluate the model performance repeated 500 times. Both resulting models, for CAMHB and HoS-CAMHB, contained each four features and achieved a satisfactory balance between model accuracy and complexity (Pareto optimality) (42). Applying this labeling logic, the overall classification accuracy was 91.5% for CAMHB (*n* = 60) and 93.5% for HoS-CAMHB (*n* = 60). When triplicate measurements were aggregated at the sample level using the median scores between biological replicates (20 samples with each media condition), performance decreased to 90.0% in CAMHB and increased to 100.0% in HoS-CAMHB (Fig. 2d). In both conditions, the measurement protocol comprised 30 min in CAMHB or HoS-CAMHB, followed by 90-min exposure to ZAB in the respective media, yielding an exceptionally fast time-to-result for an AST applicable to *A. baumannii*. In general, earlier selected features in the sequence of four selected features have a higher impact on the performance than those selected later in the sequence (Fig. 3).

Although the selected features, in terms of frequency and timeframe, currently lack a clearly defined biological interpretation, their composition aligns with previous studies: they integrate information from both early and late timepoints, and from lower as well as higher frequency ranges. While it is reasonable to assume that comparing biological states, nanomotions are reflected in different time intervals, the relationship between lower and higher frequency domains remains unclear. Lower frequency ranges are generally considered more informative (28, 39), whereas higher frequency components may serve a normalizing role. However, this hypothesis is still unproven and open to debate if higher order frequencies also harbor important information over the physiological state of the cell themselves.

In our case, all features selected by the CAMHB model include one component from a high-frequency range and one component from a low-frequency range. First, the most general selected feature is based on the Jensen–Shannon Distance (square root of divergence) (43), both of which quantify differences between probability distributions derived from the signals and a reference distribution; in essence, they measure how distinct the signals of susceptible and non-susceptible phenotypes are from a chi-squared distribution.

In the HoS-CAMHB model, half of the selected features originate from low-frequency ranges. The first and third features rely on high frequencies, whereas the second and fourth utilize low frequencies, demonstrating a more unbalanced contribution from both domains. The first and second selected features are derived from the kurtosis feature (43), which reflects how sharply the distribution concentrates around its mean relative to its tails (Table 1).

**TABLE 1:** Derived features from the quantile spectra used in the CAMHB and HoS-CAMHB models^*a*^

Feature	Measure	Statistics	1st freq (kHz)	1st time (min)	Operation	2nd freq (kHz)	2nd time (min)
CAMHB model							
F1	JS-dist	q10	4–5	30–45	Over	0.2–0.4	30–45
F2	Kurtosis	Mean	0.2–0.4	60–75	Minus	8–10	45–60
F3	Poly(deg 3) coeff 1	Slope	5–6	90–105	Over	0.1–0.2	30–45
F4	Poly(deg 3) coeff 2	q10	1–2	90–105	Minus	0–0.01	30–45
HoS-CAMHB model							
F1	Kurtosis	Mean	8–10	90–105	Minus	4–5	60–75
F2	Kurtosis	std	2–4	60–75	Over	0.1–0.2	60–75
F3	Middle-vs-out	std	4–5	45–60	Minus	5–6	30–45
F4	JS-div	Slope	0.4–1	105–120	Minus	0.1–0.2	60–75

^*a*^ JS-dist (Jensen–Shannon Distance) is the square root of the Jensen–Shannon divergence and satisfies the properties of a true metric (including the triangle inequality). It provides a normalized measure of the statistical distance between two distributions. This intuitive measure represents the distance between empirical and reference distributions; kurtosis measures the tailedness or peakedness of the empirical probability density function (ePDF). It is computed directly from the ePDF values rather than from raw signal samples, describing how sharply the distribution concentrates around its mean relative to its tails; Poly(deg3)coeff2 corresponds to the coefficient which represents the quadratic term of a third-degree polynomial fitted to the percentile sequence of the empirical distribution. It captures the degree of curvature or asymmetry in the central region of the percentile trend; Poly(deg3)coeff1 is the linear coefficient of the cubic polynomial fit that describes the general slope or monotonic trend in the percentile distribution. In practice, this parameter quantifies directional bias in how the percentile values change across their normalized positions, offering insight into the overall gradient of the empirical distribution; Middle-vs-Out represents the ratio which compares the spread of percentile values in the central portion of the distribution to that of the full percentile range. This feature quantifies the concentration of the central region relative to the total dispersion.); (JS-div (Jensen–Shannon divergence) is a measure of dissimilarity between two probability distributions. In this study, it quantifies how much the ePDF signal deviates from a reference chi-squared distribution. This metric determines how structured or random the signal is.

## DISCUSSION

This study demonstrates that nanomotion technology, when coupled with ML-based analysis, can provide rapid, growth-independent ZAB susceptibility testing for CRAB, which remains one of the most challenging pathogens due to limited treatment options and the lack of rapid diagnostic tools. Nanomotion technology has been established as a growth-independent phenotypic method for assessing antibiotic susceptibility across compounds with diverse MoA. Although phenotypic, nanomotion-based AST is influenced by the antibiotic’s MoA, and classification models consistently perform best within specific species–antibiotic combinations (e.g., Enterobacterales + ceftriaxone, *M. tuberculosis* + isoniazid, and others) (27, 33).

**Fig 4:**
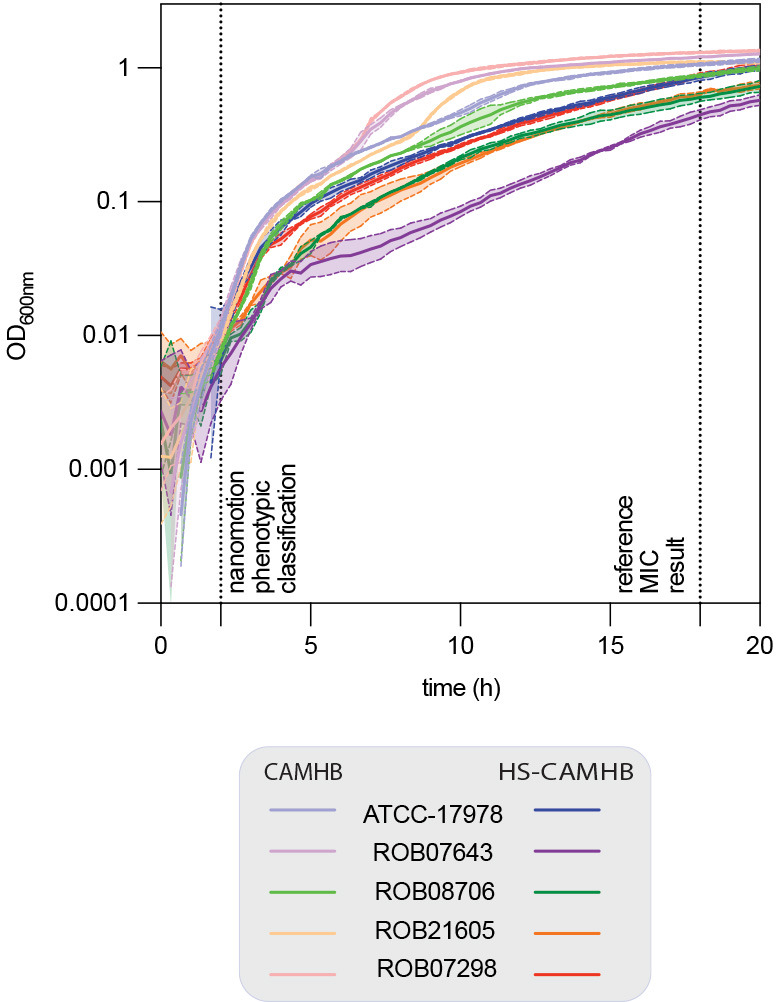
Growth kinetics of *A. baumannii* strains in CAMHB and HoS-CAMHB media. Growth curves of each strain measured over 20 h. Plotted are mean values and standard deviation from triplicate measurements. The vertical dotted line at 2 h marks the time to result for the nanomotion-based phenotypic classification, and the line at 18 h indicates the time to result for the reference AST (MIC) determination.

In our ZAB models, media composition markedly influenced feature selection and overall performance. Horse serum supplementation increases medium viscosity, which dampens cantilever oscillations, and biologically reduces *A. baumannii* growth (Fig. 4). Despite these effects, both CAMHB and HoS-CAMHB models achieved high accuracy, indicating that the nanomotion-trained algorithm predominantly captured antibiotic-specific cellular responses. The model trained in horse serum-supplemented media, however, demonstrated superior performance, particularly at the sample-aggregated level, and aligned more closely with classical MIC-based susceptibility determinations (23). The comparatively low specificity of models trained in unsupplemented media, i.e., reduced correct classification of non-susceptible responses, would be problematic in clinical decision-making and suggests that horse serum should also be included during nanomotion measurements to better mirror broth microdilution outcomes. This aligns with CLSI guidelines recommending serum supplementation for ZAB testing and underscores the robustness of this condition for phenotypic assessment (44). This study is limited by the relatively small number of strains and data points, which may affect the generalizability of the findings. The misclassifications of ROB21605, a wild-type strain with increased MIC, contributed to the drop in specificity of the CAMHB models at the sample level. Furthermore, all experiments were conducted with spiked blood cultures, and the performance of the approach in clinical settings directly from patient-derived PBCs remains to be established.

The discriminative features identified by the models reflect complex cellular responses and biophysical processes to ZAB exposure. In both media, the discriminative features comprised combinations of low- and high-frequency components, consistent with previous studies (27, 37). Features derived from high frequencies involved Jensen–Shannon distance or kurtosis and were particularly informative. In the last few years, interpretation of the nanomotion signal has made significant progress, from a simple analysis of variance changes to more complex signal interpretation, requiring signal transformation and machine learning techniques to identify changes of statistical measures in defined frequency ranges. Yet, the relation between a mathematical description, identified in this work in the form of a feature, and a biological process will require further intense study.

Currently, nanomotion AST for ZAB is qualitative, relying on reference MIC-based labeling in relation to ZAB measurement concentrations rather than defined clinical breakpoints. Developing such ultra-rapid phenotypic assays before formal breakpoint establishment can accelerate diagnostic readiness and early evaluation of new antibiotics. Once breakpoints are defined, retraining with larger isolate collections will be required to ensure robustness and generalizability.

Nanomotion analysis also revealed a cellular response preceding measurable killing. Previous studies observed CFU decline only after 2 h at 128× MIC (23) that is beyond our recording window of only 90-min drug exposure for the ML-based classification model, suggesting that nanomotion detects a pre-lethal or anticipatory response while cells remain viable. Notably, this study showed that the above-MIC concentration 0.5 µg/mL allowed the correct classification of the reference strain ATCC 17978 as susceptible phenotype whereas the time-kill kinetics did not show a killing effect, i.e., drop in CFUs, before 2 h of ZAB exposure. This indicates that nanomotion measurements capture early physiological responses to ZAB, preceding measurable decreases in bacterial viability with classical methods and thus, nanomotion signature could provide valuable insight into ZAB-induced stress in parallel to future mechanistic investigations. Clearly, a very interesting observation that should be further investigated.

In conclusion, nanomotion technology provides a rapid, growth-independent phenotypic platform for ZAB susceptibility testing against CRAB, accurately reflecting cellular responses prior to loss of viability. By providing rapid susceptibility information within 2 h, nanomotion technology may support future evidence-based antibiotic use and helps preserve the clinical utility of newly developed agents, such as ZAB. Overall, the nanomotion platform itself is a maturing and increasingly reliable phenotypic technique that has been clinically validated in Gram-negative bacteremia (36); however, the machine-learning coefficients reported here, specific to zosurabalpin, should be regarded as a proof-of-concept prototype that will require more extensive data sets for retraining and validation when applied to geographically diverse or genetically complex clinical cohorts.

## MATERIALS AND METHODS

### Bacterial strains

The *Acinetobacter baumannii* ATCC 17978 reference strain (MIC = 0.06 µg/mL) was acquired from LGC. The three wild-type isolates ROB07643 (MIC = 0.03 µg/mL), ROB08706 (MIC = 0.5 µg/mL or MIC = 1 µg/mL in the presence of 20% heat-inactivated horse serum), ROB21605 (MIC = 4 µg/mL), and the laboratory-generated *lptF* mutant ROB07298 (MIC > 64 µg/mL) were obtained from F. Hoffmann-La Roche, Basel, Switzerland. Strains were stored at −80°C in 20% glycerol.

As a control strain, *Escherichia coli* ATCC 25922 was used (ceftriaxone MIC = 0.047 µg/mL; meropenem MIC = 0.016 µg/mL).

### Generating spiked blood cultures

Bacterial samples for nanomotion experiments were prepared by culturing the strains on non-selective agar plates at 37°C overnight, single colonies were resuspended in 0.9% NaCl (PanReac AppliChem) to achieve a 0.5 McFarland density. Two 1:10 serial dilutions were followed by the transfer of 10 µL of the final dilution to 9,990 µL of EDTA blood, obtained from a Swiss blood donation center, fully anonymized. The 10 mL of artificially infected blood was inoculated with a syringe in aerobic (AEH) blood culture bottles (BD BACTEC Standard Aerobic medium Culture Vials; Becton Dickinson). Spiked blood cultures were incubated in the BACTEC 9240 automated blood culture system (Becton Dickinson) until identified positive for bacterial growth (around 11 h or overnight).

### Cantilever sensor and cantilever functionalization

The quartz-like cantilevers (NanoWorld AG, Switzerland) contain a gold-coated tip that acts as a mirror for the light beam (spring constant: 0.1 N/m, dimensions: 130 × 40 × 0.75 µm, resonant frequency: 32 kHz). They act as sensors for the bacterial nanoscale vibrations, oscillating at specific frequencies. No absolute calibration of the cantilevers (e.g., thermal tuning or force–distance curves) was performed to standardize sensitivity across experiments. The classification does not depend on absolute deflection amplitudes: the variance is normalized by the total detected signal (SUM; see below), and the model features are relative, quantile-based descriptors of the power spectral density derived within each experiment (see Table 1). This relative feature-extraction architecture therefore makes the approach largely insensitive to absolute nanometric calibration and to cantilever-to-cantilever differences in absolute sensitivity.

To immobilize bacteria on the cantilever during AST recording, the cantilever was incubated with 50 μL of 0.1 mg/mL PDL (PDL, Gibco) for 20 min at room temperature (RT). Then, the PDL drop was removed and discarded, and the cantilever was gently washed with 100 μL of molecular biology-grade water (HyClone). They were let dry for at least 15 min before use and kept dry for up to 6 months. Cantilevers stored dry at room temperature for up to ~6 months showed no apparent difference in initial bacterial attachment density, attachment kinetics, or baseline sensor signal (blank-phase variance) compared with freshly functionalized cantilevers, indicating that the surface functionalization remained stable over this storage period. This treatment created a positive electric charge that facilitated the attachment of negatively charged bacteria.

### Attaching bacteria to the cantilever and nanomotion-based AST conditions

The bacterial pellets for nanomotion measurements were prepared following the MBT Sepsityper IVD Kit (Bruker) instructions. Briefly, 1 mL of blood culture was mixed with 200 µL of lysis buffer, vortexed, and centrifuged at 12,000 × *g* for 2 min. The bacterial pellet was resuspended in 1 mL of washing buffer, then centrifuged again for 1 min at 12,000 × *g* to remove debris. The pellets were resuspended in 200 µL 1× PBS (Corning) containing 0.04% (w/v) agarose. The bacterial suspension was incubated on the cantilever on Parafilm M (Amcor) for 1–3 min at room temperature to immobilize the cells. Then, a gentle PBS wash was performed to remove unattached bacteria, and the attachment quality was assessed by phase microscopy prior to placing the sensor in the Resistell Phenotech device (Resistell AG, Muttenz, Switzerland) for nanomotion measurements. In case of unsatisfactory attachment, the incubation process was repeated.

The Resistell Phenotech device consists of a stainless-steel head with a separated fluid measurement chamber, which can be independently positioned for sample handling. The set-up includes an active vibration damping system, custom acquisition and control electronics, and a dedicated computer terminal. Its cantilever-based optical deflection system differs from standard atomic-force microscopy (AFM) setups, as it positions the light source and photodetector below the cantilever, simplifying the manipulation. A light beam (super-luminescent diode or SLED, 650 nm, 2 mW) is focused on the tip of the cantilever, whose reflective surface acts as a mirror and redirects the light onto a four-sectional position-sensitive photodetector. The cantilever deflection signals are converted into electrical signals via custom electronics (Resistell AG) and stored in a data acquisition card (USB-6212; National Instruments, USA), controlled by a dedicated AST software. The head is mounted on the vibration isolation system, and both are placed inside an incubator to enable the measurements at 37°C.

### Nanomotion-based AST experiments

The nanomotion-based AST experiments consisted of three consecutive phases: a 10-min blank phase, a 30-min medium phase, and a 90-min or 8-h drug phase. During the blank phase, a new cantilever (without immobilized cells) was measured in cation-adjusted Mueller–Hinton broth (CAMHB), with or without 20% heat-inactivated horse serum (HoS-CAMHB), to record background vibrations. This step served as a quality control to detect potential cantilever malfunctions or medium contamination. A variance >2.6 × 10⁻⁶ indicated possible contamination by floating particles or external noise that could generate artifact signals. The medium phase measured the nanomotion signal after bacterial immobilization on the cantilever surface. It reflects the intrinsic nanomotions of the immobilized cells and allows them to adapt to the new conditions of medium and temperature after isolation from the original sample. Finally, during the drug phase, the real-time nanomotion response was recorded upon addition of the corresponding zosurabalpin (ZAB, Hoffmann-La Roche, Switzerland), ceftriaxone (CRO, ceftriaxone, disodium salt hemi(heptahydrate) analytical standard, Sigma-Aldrich) or meropenem (MEM, meropenem trihydrate, Sigma-Aldrich) concentration to the medium in the chamber. The signal patterns observed in this phase were later used to develop phenotypic classification algorithms. In this study, we tested different concentrations of ZAB: 0.016, 0.5, 2, and 16 µg/mL; CRO: 0.01, 0.4, and 12.5 µg/mL; MEM: 0.004, 0.128, and 4.2 µg/mL; and no drug conditions (0 µg/mL).

### Signal processing and calculation of variance and variance exponential slope

Signal acquisition was performed by recording the nanomotion in the time domain at a sampling rate of 60 kHz, converting the continuous waveform into a discrete sequence suitable for computational processing. The nanomotion signal was split into 10-s timeframes, and a running median with a 1-min time window simplifies the interpretation by illustrating how strongly the signal fluctuates around zero.

The variance (V^2^/V^2^) reflects the cantilever oscillation amplitude. It is calculated as the ratio between the cantilever deflection (detected in volts) and the total voltage detected (SUM). Variance plots were mainly used for the initial visual inspection of the results, as the temporal changes in the variance may indicate bacterial responses to the antibiotic. Although this variance-based analysis provides useful information on the overall level of bacterial activity, it was not sufficient on its own for reliable classification of bacterial signals. We obtained the exponential slope with the formula log(*x*)  = log(*C*) + *at*, where *t* is time (in min), *a* is the slope of the common logarithm of the variance trend, and log(*C*) is the intercept.

### Calculation of raw quantile features

After sampling, the signal is divided into overlapping time segments to minimize boundary effects, preserve signal continuity, and improve the reliability of the spectral analysis. Each segment was first detrended to remove low-frequency drift, and then a Fourier-based periodogram was computed to estimate the power spectral density (PSD), describing the distribution of signal power across frequencies and highlighting dominant components. To capture finer spectral characteristics, quantiles of spectral values were calculated across all periodograms within a selected frequency range [fmin, fmax]. These quantiles (e.g., Q10–Q90) provided a statistical description of the spectrum beyond mean-based metrics, offering insights into its distributional structure. The resulting data were assembled into a two-dimensional quantile map, where the horizontal axis represents frequency bins, and the vertical axis denotes quantile levels. Each point in the map corresponds to the spectral quantile at a given frequency. This visualization highlights both variability and distributional features of the PSD, enabling a more detailed interpretation of spectral properties.

### Calculation of derived features

Quantile-based spectral maps provide insight not only into how power spectral density (PSD) is distributed across frequency bands but also into how this distribution changes over time. To capture temporal dynamics, quantile maps were generated for successive five-minute segments within the 90-min recording of the *Drug* phase recording, effectively adding a time dimension that enables tracking of spectral variability throughout the experiment. From the PSD quantiles, a range of statistical measures were derived. In particular, differences and ratios between selected quantiles were calculated to quantify distributional properties such as kurtosis and skewness, along with other shape-related descriptors. To assess longer-term patterns, these indicators were averaged over six consecutive 15-min windows. Differences and ratios of these averages were then computed to characterize the pace, direction, and stability of spectral changes over extended timescales. This procedure yielded a set of dynamic features that jointly captured both short-term fluctuations and broader temporal trends. The resulting multi-dimensional feature space, representing the temporal evolution of quantile maps, was then used as input for machine learning models. By integrating short- and long-term spectral information, these features provided a robust foundation for classification tasks.

### Feature selection

A feature selection procedure was applied to identify features most relevant to the phenomenon under study. From the feature space, those that maximized the classifier’s performance were retained. The model is a classifier, whose effectiveness was evaluated using accuracy as the main metric to assess its ability to discriminate between susceptible and non-susceptible groups. Feature selection was performed using a forward selection strategy reinforced by backward selection strategy. In this iterative process, each feature was evaluated within the classifier using repeated stratified cross-validation. The feature yielding the greatest improvement in accuracy was added to the selected set and removed from the remaining pool of candidates. The process was then repeated, and the remaining features were tested in combination with those already selected, and the best-performing one was added in each iteration. During each iteration, the five best-performing features were retained and passed to the backward feature selection algorithm. From these sets, all possible feature combinations were generated by selecting one feature from each iteration’s top set. Each combination was then evaluated using repeated stratified cross-validation to identify the best-performing collection of features. The feature selection process was completed after seven iterations. Cross-validation results indicated that a subset of four features achieved a satisfactory balance between model accuracy and complexity, minimizing the risk of overfitting.

### Classification model development

Logistic regression models the relationship between predictor variables and binary classes. It relies on the logistic function to map the output of a linear combination of predictors into a floating-point value. Classification is then performed using a threshold of 0, assigning observations above it to one category and below it to the other (in this case, susceptible or non-susceptible group). The absolute value of a decision function score reflects the strength of each predictor’s contribution to the likelihood of a sample belonging to a given class. This approach is straightforward to interpret, allowing insight into the model’s decision-making process, in contrast to more complex models such as multilayer perceptrons.

### Metrics and scores

To obtain reliable performance metrics, the model was tested using cross-validation, a technique used to evaluate the performance and generalization ability of a machine learning model. Instead of relying on a single split of training and testing data, the data set is partitioned into multiple subsets, referred to as folds. In each iteration, one fold is reserved for tests, while the remaining folds are used for training. This process is repeated until every fold has served as a test set. In this study, repeated stratified k-fold cross-validation was used, where the data are divided into *k* equally sized folds with a similar distribution of artificial labels. Model performance is then assessed across all *k* iterations, and the results are averaged to provide a robust estimate of accuracy and stability. The procedure was repeated 500 times over threefold. In order to assign one decision function score to each experiment, the 500 scores produced by cross-validation were aggregated by calculating the mean of scores. Then, the performance metrics of the cross-validation per experiment were obtained by calculating accuracy, specificity, and sensitivity of the predicted class indicated by the mean scores against the given artificial label (susceptible or non-susceptible). Finally, sample-level metrics and scores were obtained by taking the median scores across the biological replicates for each strain and ZAB concentration.

### Time-kill kinetic determination

In parallel to the 8-h drug phase nanomotion AST recordings, and starting from the same PBC, the bacterial pellet was extracted with the MBT Sepsityper IVD Kit (Bruker) and resuspended in 200 µL of 1× PBS. Then, 10^6^ CFU/mL were inoculated into six tubes containing 2 mL HoS-CAMHB and incubated at 37°C without shaking to mimic a 30-min medium phase, followed by the addition of different concentrations of ZAB in each tube (0, 0.016, 0.06, 0.5, 2, 16 µg/mL).

The kinetics of killing were determined by sampling (30 µL at each timepoint: 0, 1, 2, 4, 6, and 8 h), plating the corresponding sample dilutions on non-selective agar plates, overnight incubation, and CFU counting for each ZAB concentration. The experiment was performed in triplicates, and the results were plotted using GraphPad Prism.

### Growth curve

We assessed the growth rate of each bacterial strain in this study in both CAMHB and HoS-CAMHB media. The growth of 1 × 10^6^ CFU/mL of each isolate coming from an exponential CAMHB liquid culture was monitored in both media. OD_600_ measurements were done every 20 min for 20 h at 37°C, shaking for 5 s before every measurement with 200 rpm, using a 96-well plate and the FLUOstar Omega plate reader (BMG Labtech). Analyses were done with blank correction for each medium and calculating the mean and standard deviation of the triplicate measurements for each strain and medium.

## Data Availability

The data sets generated and analyzed during the current study, including the nanomotion recordings and derived feature data, are available from the corresponding author (A.S.) upon reasonable request. The code used for signal processing, feature extraction, and the logistic-regression classification model is also available from the corresponding author upon reasonable request.

## SUPPLEMENTAL MATERIAL

Figure S1 spectrum.04064-25-s0001.pdf *A. baumannii* biomass stability on cantilevers during nanomotion recordings and nanomotion recordings of *E. coli*.

ASM does not own the copyrights to Supplemental Material that may be linked to, or accessed through, an article. The authors have granted ASM a non-exclusive, world-wide license to publish the Supplemental Material files. Please contact the corresponding author directly for reuse.
