# Supplementary material for: Rapid delineation of Acinetobacter baumannii dose responses to zosurabalpin based on machine learning-assisted interpretation of bacterial nanomotions
Source: Microbiol Spectr. 2026 Jun 30;14(8):e04064-25. doi: 10.1128/spectrum.04064-25 (PMC13435977; doi:10.1128/spectrum.04064-25)
Supplement: Supplementary file 1 [file spectrum.04064-25-s0001.pdf]

Supplementary Information

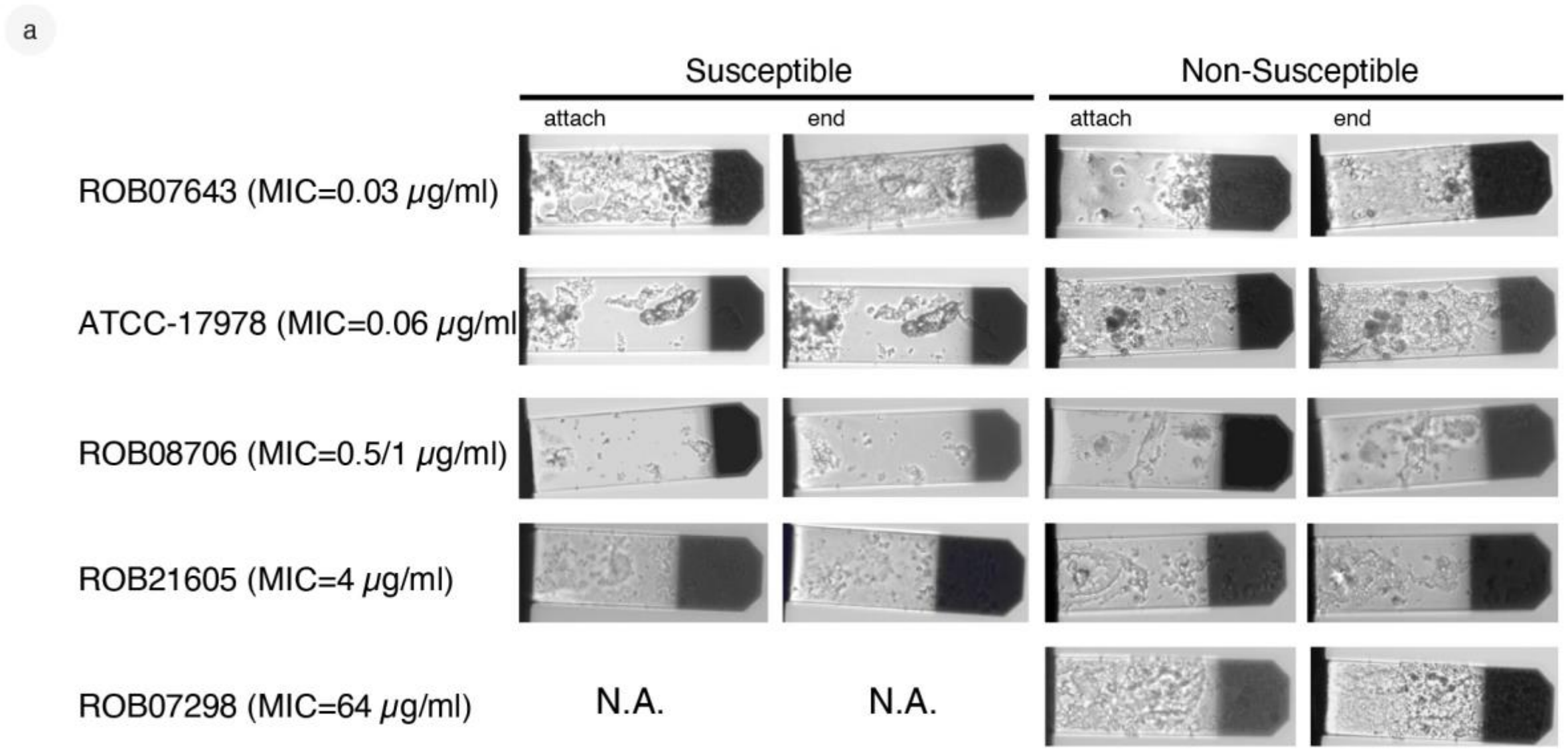

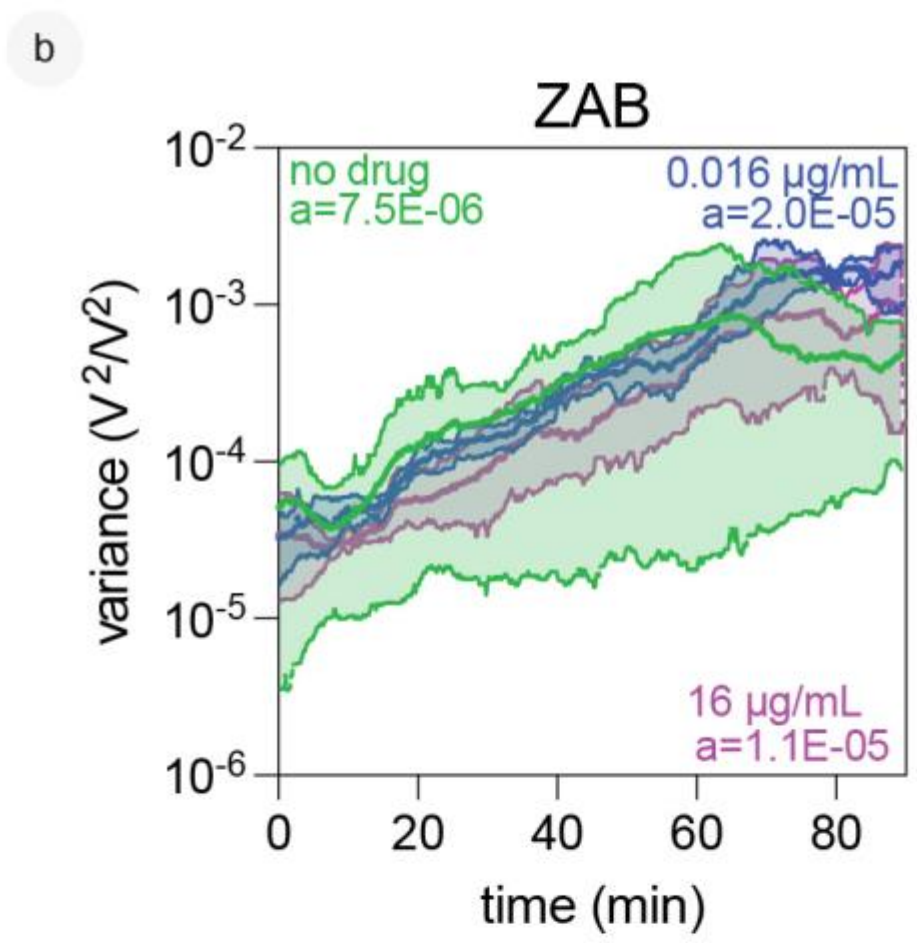

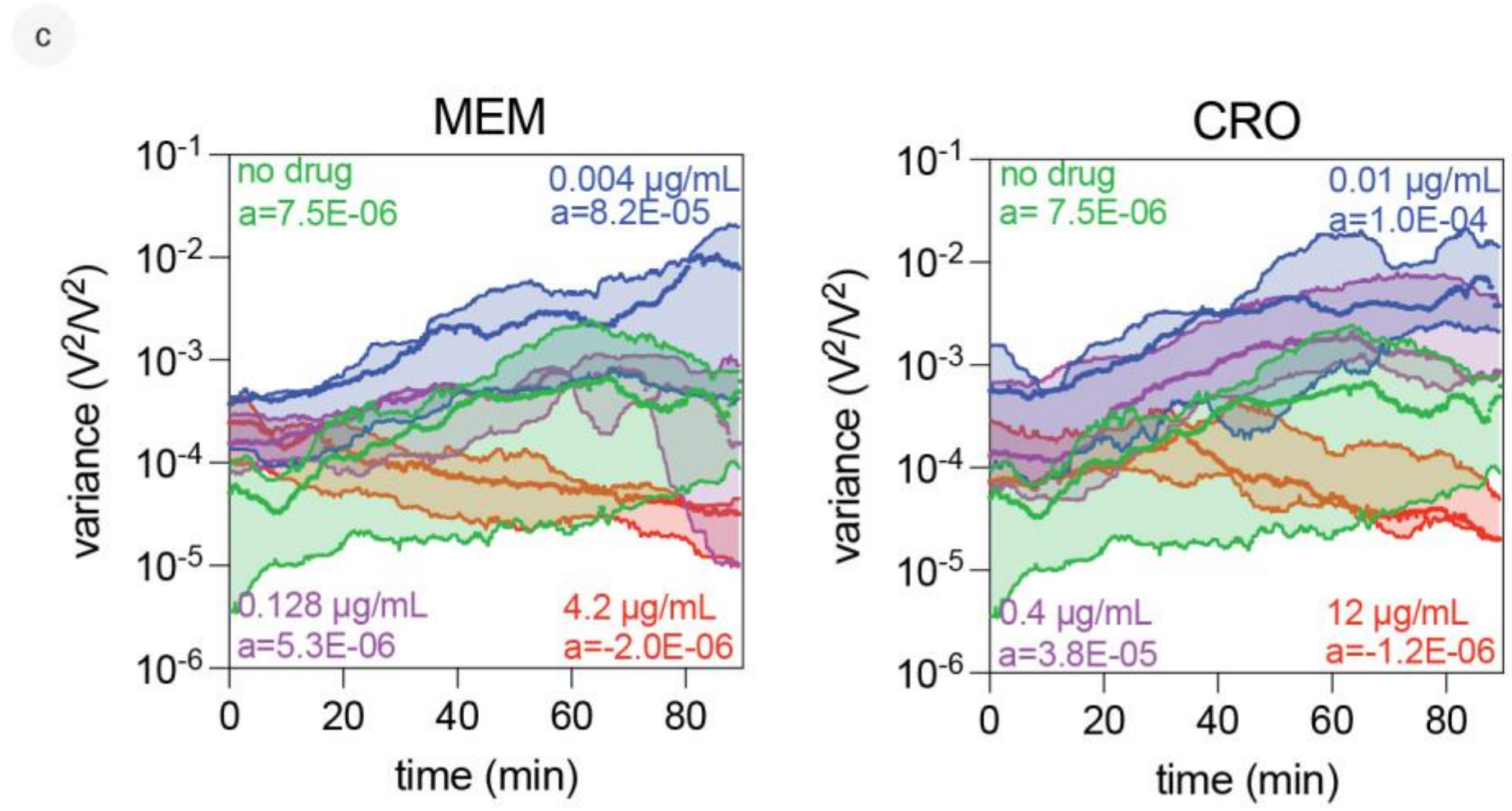

Supplementary Figure 1 | *A. baumannii* biomass stability on cantilevers during nanomotion recordings and nanomotion recording of *E. coli*.

a) Representative microscopy images of the five tested strains under susceptible (S) and non-susceptible (NS) conditions acquired immediately after bacterial attachment to the cantilever, prior to nanomotion recording (attach), and at the end of the experiment (end). Only minimal changes in biomass were observed throughout the recording period, including under NS conditions.

b) Nanomotion responses of *E. coli* ATCC 25922 exposed to antibiotics with distinct activity spectra and pharmacodynamic properties, nanomotion variance plots of *E. coli* ATCC 25922 exposed to zosurabalpin (ZAB) at three concentrations. No significant alterations in nanomotion variance were observed under any tested condition, consistent with the reported *A. baumannii*-specific activity of ZAB.

c) Nanomotion variance plots of *E. coli* ATCC 25922 exposed to ceftriaxone (CRO) and meropenem (MEM), respectively. Although both antibiotics belong to the β-lactam class, they represent distinct subclasses with different pharmacodynamic properties and killing kinetics. Distinct temporal nanomotion patterns were observed following exposure to CRO and MEM, highlighting the strong influence of antibiotic-induced cellular effects on nanomotion responses. All concentrations were selected to match the same MIC-relative ratios used throughout the study (0.25×, 8×, and 267× MIC), corresponding to ZAB 0.016, 0.5 and 16 µg/mL; CRO 0.01, 0.4 and 12.5 µg/mL; and MEM 0.004, 0.128 and 4.2 µg/mL. Each condition was tested in triplicate.
